# Phenolic Constituents of *Chrysophyllum oliviforme* L. Leaf Down-Regulate TGF-*β* Expression and Ameliorate CCl_4_-Induced Liver Fibrosis: Evidence from In Vivo and In Silico Studies

**DOI:** 10.3390/antiox8120646

**Published:** 2019-12-15

**Authors:** Seham S. El-Din El-Hawary, Soheir M. El Zalabani, Nabil M. Selim, Marwa A. Ibrahim, Fatma A. Wahba, Shymaa A. El Badawy, Nariman El Sayed Mahdy, Aziz Yasri, Mansour Sobeh

**Affiliations:** 1 Department of Pharmacognosy, Faculty of Pharmacy, Cairo University, Cairo 12613, Egypt; seham.elhawary@yahoo.com (S.S.E.-D.E.-H.); selzalabani@gmail.com (S.M.E.Z.); narimanmahdy@yahoo.com (N.E.S.M.); 2 Department of Biochemistry, Faculty of Veterinary Medicine, Cairo University, Cairo 12613, Egypt; marwa199@gmail.com; 3 Department of Physiology, Faculty of Veterinary Medicine, Cairo University, Cairo 12613, Egypt; F_wahba@hotmail.com; 4 Department of Pharmacology, Faculty of Veterinary Medicine, Cairo University, Cairo 12613, Egypt; 5 AgroBioSciences Research Division, Mohammed VI Polytechnic University, Lot 660–Hay MoulayRachid, Ben-Guerir 43150, Morocco; aziz.yasri@um6p.ma

**Keywords:** *Chrysophyllum oliviforme*, *Chrysohyllum cainito*, hepatoprotective, antioxidant, TGF-β, phenolic compounds

## Abstract

The prevalence of hepatic diseases globally and in Egypt particularly necessitates an intensive search for natural hepatoprotective candidates. Despite the traditional use of *Chrysophyllum oliviforme* L. and *C. cainito* L. leaves in the treatment of certain ailments, evidence-based reports on their bioactivities are limited. In this work, in vivo and in silico studies were conducted to evaluate their methanol extracts potential to alleviate liver damage in CCl_4_-intoxicated rats, in addition to their antioxidant activity and identifying the molecular mechanisms of their phenolic constituents. The extracts restored the altered total cholesterol (TC), triglycerides (TG), high-density lipoproteins (HDL), alanine aminotransferase ALT, aspartate aminotransferase AST, total protein, and albumin. Histopathological architecture, DNA fragmentation, and mRNA expression level of TGF-β1 also confirmed the anti-fibrotic activity of the two extracts. The total phenolic content (TPC) in *C. oliviforme* ethanol extract exceeded that in *C. caimito*. Additionally, the malondialdehyde (MDA), reduced glutathione (GSH), and total antioxidant capacity (TAC) levels assured the antioxidant potential. Seven phenolics; quercetin, isoquercitrin, myricetin, kaempferol, and caffeic, trans-ferulic, and gallic acids were isolated from the ethanol extract of *C. oliviforme*. The molecular docking of isolated compounds revealed a low binding energy (kcal/mol with TGF-β1, thus confirming the hepatoprotctive activity of the extracts. In conclusion, the *C. oliviforme* leaves could be considered as potent safe raw material for the production of herbal formulations to alleviate hepatic toxicity after preclinical safety study.

## 1. Introduction

Oxidative chain reactions induce cellular damage and homeostatic disruption, causing a wide number of health problems, among them liver injury. The distortion of the normal liver assembly with excessive accumulation of extracellular matrix distinguishes the latter [1]. Hepatocyte damage promotes the secretion of inflammatory and profibrogenic cytokines, leading to the development of liver fibrosis. The transforming growth factor-β (TGF-β1) is the most potent profibrogenic cytokine that regulates the proliferation and differentiation of many cell types by directing the expression of hundreds of target genes [2]. In the hepatic stellate cells (HSCs) of liver, for instance, TGF-β1 stimulates the activation of SMADs (main signal transducers for receptors of TGF-B) and mitogen-activated protein kinases (MAPKs) signaling pathways [3]. Antioxidant compounds, among them plant polyphenols, can prevent cellular injury by inhibiting the initiation or propagation of such oxidative reactions [4].

*Chrysophyllum* L. species (Sapotaceae) are widely propagated as ornamentals due to their dense colorful foliage and for their edible fruits. Among these, *C. oliviforme* L. (Satin leaf) and *C. cainito* L. (Golden leaf tree) are successfully acclimatized in Egypt. The occurrence of triterpenoids and sterols has been reported from the leaves of *C. cainito* [5,6], besides several polyphenols that were isolated from its edible fruits [7,8]. On the other hand, the chemical composition of *C. oliviforme* is not, by far, thoroughly identified. In addition, the few some available biological screening studies have restricted the bioactivities of the plant to an in vitro evaluation of its antioxidant and cytotoxic potential [9].

There are various drugs that can induce liver injury in animal model; among them are antibiotics (Tetracycline) and analgesics and antipyretic drugs (Acetaminophen), as well as, animal model of drug induced liver injury (DILI), which is still under clinical challenge, because of the low predictability of result. Other models have been fully established while using hepatotoxicants as CCl_4_ and galactosamine or acetaminophen overdose. CCl_4_ overdose causes a reproducible acute liver injury that resembles intrinsic (DILI) [10]. The repetitive administration of CCl_4_ is a classical method of inducing liver fibrosis. The examination of gene expression revealed significantly less proliferative response after galactosamine injury than that of CCl_4_. Moreover, the timing of proto-oncogene expression during liver regeneration might vary considerably after thioacetamide. It has been observed that the histopathology of CCl_4_ toxicity in rats is like that in humans [11].

In this study, we explored the hepatoprotective and anti-fibrotic activities of both *C. cainito* L. and *C. oliviforme* L. against CCl_4_-induced liver damage. Additionally, we isolated and characterized the chemical constituents of the most active extract from *C. oliviforme*. Furthermore, the interaction of the isolated compounds with TGF-β utilizing molecular docking tools confirmed the hepatoprotective properties of the extract.

## 2. Materials and Methods

### 2.1. Plant Material

Fresh leaves of *C. oliviforme* L. and *C. cainito* L. (Sapotaceae) were collected during Spring season (April–May 2015) from plants that were cultivated at the Zoological garden (Giza, Egypt). The taxonomic identity was confirmed Voucher specimens (#2015.04.06 I and 2015.04.06 II) were kept at the herbarium of the Pharmacognosy Department, Faculty of Pharmacy, Cairo University, Giza, Egypt. The leaves were dried under shade and finely powdered to give 2 kg each.

### 2.2. Plant Extracts and Sample Solutions

Powdered leaf samples (2 kg, each) were exhaustively extracted with petroleum ether (b.r. 40–60 C, 3 × 6 L), followed by ethanol (70%, 8 × 6 L). The solvents were distilled under vacuum, in a rotary evaporator (Büchi, Flawil, Switzerland). The dried petroleum ether and ethanol extracts of *C. oliviforme* (encoded Op and Oe) and *C. cainito* (encoded Cp and Ce) were weighed and their percentage yields were calculated. The petroleum ether and ethanol 70% extracts of *C. oliviforme* were obtained in higher yields than those of *C. cainito*, corresponding to 8.45% and 10% vs 6.8% and 8.01% of dried plant material, respectively.

### 2.3. Experimental Animals and Laboratory Diet

The Institutional Animal Care and Use Committee of Cairo University (CU-IACUC) approved the experimental protocol, approval code; CU III S 79 17. Adult male Wistar rats were used for their higher sensitivity to CCl_4_ toxicity than females [12]. Rats aged (8–10 weeks) and weighing (160–190 g) were obtained from the Animal House Colony at Vac-Sera, Egypt, and then housed in laboratory animal housing facilities at the Faculty of Veterinary Medicine, Cairo University, Giza, Egypt. The animals were allowed to acclimatize for one week before starting the experiment and maintained under standard laboratory conditions (controlled room temperature, 22 ± 1 °C; relative humidity, 54–68%, and 12 h light/dark cycle) with free access to a well-balanced diet (vitamins mixture, 1%; minerals mixture, 4%; corn oil, 10%; sucrose, 20%; cellulose, 0.2%; casein, 10.5%; and, starch, 54.3%) and water. 

### 2.4. Drugs and Diagnostic Kits

Silymarin (Sedico Pharmaceutical Co., 6th October City, Egypt) served as standard hepatoprotective reference drug. Biochemical kits for measuring lipid profile, liver function and oxidative stress parameters; total cholesterol (TC) (Cat. No. 230002), triglycerides (TG) (Cat. No. 314001), high-density lipoproteins (HDL) (Cat. No. 266001), total protein (Cat. No. 310001), albumin (Cat. No. 211001), aspartate aminotransferase AST (Cat. No. 260002), alanine aminotransferase ALT (Cat. No. 292002), MDA (Cat. No. MD 25 29), reduced glutathione (GSH) (Cat. No. GR 25 11), and total antioxidant capacity (TAC) (Cat. No. TA 25 13)) were purchased from Biodiagnostic Co. (Dokki, Giza, Egypt). RNeasy Mini Kit (Qiagen, Cat. No. 74104, USA), Revert Aid First Strand cDNA Synthesis Kit (Cat. No. 1622), and Luminaries Color HiGreen Low ROX qPCR Master kit (Cat. No. K0973) (Thermo Scientific, Waltham, MA, USA) were used for genetic studies of isolated livers.

### 2.5. Chemicals and Chromatographic Materials

All of the chemicals used in the study were of analytical grade. Carbon tetrachloride (Cat. No. 319961, Sigma-Aldrich, St. Louis, MO, USA) was dissolved in normal saline and then used for the induction of hepatotoxicity. Silica gel H60 (Cat. No. 107735, Merck, Darmstadt, Germany). Silica gel RP-18 (Cat. No. 60757, 70-230 mesh, Sigma-Aldrich, Germany) and Sephadex LH-20 (OCLC NO. 35265084, Pharmacia Fine Chemicals AB, Uppsala, Sweden) were used for column chromatography, and pre-coated silica gel 60F 254 (Fluka, Sigma-Aldrich Chemicals, Germany) was used for TLC (thin layer chromatography). The reference phenolic compounds were purchased from Sigma-Aldrich (St. Louis, MO, USA).

### 2.6. Evaluation of Hepatoprotective and Antioxidant Activities

#### 2.6.1. Experimental Design

Seventy-seven rats were randomly allocated into 11 groups of seven rats each. The animals of group I, serving as negative control, received a daily intragastric oral dose of (1 mL) normal saline with the vehicle (1% methylcellulose) for six weeks. Animals of groups II-XI received orally while using gavage the different samples; vehicle (1% methylcellulose), standard and extracts, for six weeks, before induction of hepatic toxicity through subcutaneous injection of 1:1 *v/v* mixture of CCl_4_ and liquid paraffin at a dose of 1 mL/kg b.wt., every 72 h, for seven days. During the whole study period, Group II (positive control) only received the vehicle (1% methylcellulose) and Group III received daily oral dose of (100 mg/kg b.wt.) Silymarin (standard hepatoprotective) [13]. Groups (IV–VII) were treated with the petroleum ether and defatted ethanolic extracts of *C. canito*, each at two doses of 250 and 500 mg/kg b.wt, respectively. Groups (VIII–XI) were treated with the petroleum ether and defatted ethanolic extracts of *C. oliviforme*, each at two doses of 250 and 500 mg/kg b.wt, respectively. The blood samples were collected from the tail vein under anesthesia with ketamine at dose of 91 mg/kg in duplicates, which were collected into plain and heparinized tubes. The latter samples were used for antioxidant assays. The sera were separated by blood centrifugation at 3000 rpm at 4 ℃ for 10 min. and then used for different biochemical assays. The specimens of liver tissues were used for histopathological examination, after cervical dislocation of animals, and 100 mg portions were dissected and separately stored at −80 °C to be used for the genetic study.

#### 2.6.2. Biological Assays

The lipid profile and liver function parameters (TC, TG, HDL, total protein, albumin, AST, and ALT) were estimated by means of a diagnostic kit according to previously described methods; total cholesterol (TC) [14], triglyceride concentrations (TG) [15], high density lipoprotein (HDL) [16], alanine and aspartate aminotransferase (ALT and AST) [17], total protein [18], and albumin concentration [19].

#### 2.6.3. Antioxidant Assay

The heparinized whole blood samples were used for the spectrophotometric estimation of glutathione activity (GSH); with method based on the reduction of 5,5’ dithiobis (2- nitrobenzoic acid) (DTNB) with glutathione [20]. Lipid peroxide (malondialdehyde, MDA, formation) was spectrophotometrically estimated while using heparinized plasma and the method based on Thiobarbituric acid (TBA) reacted with malondialdehyde (MDA) in acidic medium at temperature of 95 °C for 30 minlipid peroxidation [21]. Total antioxidant capacity (TAC) was measured while using the reaction of antioxidants in the sample with a defined amount of exogenously provide hydrogen peroxide and total antioxidant capacity (TAC) [22]. 

### 2.7. Histopathological Examination

Paraffin sections (2 mm thick) of buffered formalin-fixed liver samples were immediately prepared, after decapitation. The extent of CCl_4_-induced necrosis was evaluated by assessing morphological changes in liver sections stained with both hematoxylin and eosin (H & E) [23] and Masson’s trichrome (MTC) while using an appropriate kit (Sigma-Aldrich Co. LLC, St. Louis, MO, USA), according to the manufacturer’s instructions. An experienced histologist, blinded to the examined samples identity to avoid any bias, performed histopathological processing and the assessment of specimens. 10 microscopic fields per section (seven sections per group one per animal condition) were examined by light microscope (Olympus BX50, Tokyo, Japan). 

### 2.8. Genetic Profiling of Liver Tissues

#### 2.8.1. Real Time Reverse Transcriptase—PCR

Two techniques viz., DNA laddering assay and measurement of mRNA expression level of TGF-β, the latter was measured by real-time PCR, assessed the extent of amelioration in genetic profiles of damaged liver tissue, gained upon administration of leaf extracts of the two plants. Total RNA was extracted from fresh liver tissues using RNeasy Mini Kit, according to the standard protocol. Its concentration and purity were checked by UV absorption, at λ_max_ 260 and 280 nm, respectively, while using a NanoDrop spectrophotomer (Thermo Scientific, Waltham, MA, USA). The first-strand cDNA was synthesized by means of Revert Aid First Strand cDNA Synthesis Kit. The target mRNA expression was quantified while using the Luminaries Color HiGreen Low ROX qPCR Master Kit. Real-time PCR analysis was carried out under the following conditions: holding stage 95 °C, 30s; cycling stage 95 °C, 5s, 60 °C, 30s (45 cycles); and, melt curve stage: 95 °C, 15s; 60 °C, 2 min; 95 °C, 15s. β-actin was used as the internal control. The primer sequences for TGF-β were forward: 5’GGACTCTCCACCTGCAAGAC-3’ and reverse 5’CTCTGCAGGCGCAGCTCTG-3’ [24]. The m-RNA levels were measured in terms of cycle threshold (Ct), followed by normalization to the expression level of β-actin, while using the 2^−ΔΔCt^ method. The inhibition of the up-regulated expression of TGF-β in liver-damaged tissue indicated the efficiency of the drug in reducing liver fibrosis.

#### 2.8.2. DNA Fragmentation Assay

The DNA laddering assay was carried out by adopting the DPA (diphenylamine) method [25]. DNA fragments that were isolated from treated-liver tissues were subjected to electrophoresis on 2% agarose gel. The amelioration of the markedly elevated DNA fragmentation percentage, resulting from CCl_4_-intoxication, was considered as a measure of the drug ability to attenuate liver fibrosis. 

### 2.9. Phenolic Composition 

The ethanol extracts of the investigated leaves were selected for this study based on their positive response to tests for phenolic components, including flavonoids, which were carried out according to published procedures [26].

#### 2.9.1. Determination of Total Phenolic and Flavonoid Contents (TPC and TFC) 

Spectrophotometric determinations of TPC and TFC were carried out by the Folin-Ciocalteu [27] and AlCl_3_ colorimetric methods [28], respectively. The produced color absorbance was recorded for TPC and TFC at λ_max_ 765 nm and 415 nm, respectively, on a Shimadzu double beam spectrophotometer (UV-1650, Shimadzu, Tokyo, Japan). Gallic acid was used as the standard for TPC. The concentrations of phenolic compounds were expressed as μg GAE/mg dried extract, while flavonoid concentrations were expressed as μg QE/mg dried extract.

#### 2.9.2. Isolation and Identification of Phenolic Components

Ethanol extracts, which were suspended in distilled water, were subjected to successive fractionation with solvents of increasing polarity; methylene chloride, ethyl acetate; and, n-butanol saturated with water. Thirty grams of the ethyl acetate extract of *C. oliviforme* was subjected to VLC (vacuum liquid chromatography) packed with (Silica gel H 60, 500 g). Gradient elution with methylene chloride, ethyl acetate, and 100% methanol was performed. Fractions (300 mL, each) were collected and monitored by TLC (thin layer chromatography) while using solvent systems S1; methylene chloride-methanol-formic acid 85:15:0.2 *v/v* and S2; ethyl acetate-methanol-water-formic acid, 100:16.5:13.5:0.2 *v/v*. The spots were monitored by UV 365 nm and then sprayed with AlCl_3_ and/or FeCl_3_ reagents. Further purification of the selected fractions on Sephadex LH-20 and Silica gel RP-18 (reversed phase) columns were performed. The isolated compounds were identified based on physicochemical and spectral data (m.p., m.m.p. co-TLC, UV, ^1^H NMR, and ^13^C NMR). An electro thermal IA9100 apparatus (Burlington, NJ, USA) was used for the determination of melting points and a Bruker NMR-spectrometer 400 MHz (Bruker, Yokohama, Japan) for running ^1^H NMR and ^13^C NMR spectroscopy.

### 2.10. Molecular Docking of the Isolated Compounds in TGF-β Active Site

The chemical structure of the isolated compounds and silibinin (Standard hepatoprotective drug) was downloaded from ZINC Database [29] as the mol2 format and the three-dimensional (3D) structure of the transforming growth factor beta receptor type 1 (TGF-β R-1) was downloaded from Protein Data Bank (PDB: 1vjy) [30], which was loaded in the protein preparation module integrated in IGEMDOCK software for virtual screening [31]. The active site was defined by the co-crystallized ligand as sphere with radius 8A.

The compounds were loaded to the IGEMDOCK preparation module and then docked to the active site. The docking parameters were set to be as the following: Population size 300, Generations 80, and number of solutions was set to 10. Post-docking analysis was done to determine best poses and types of interaction between the compound and the binding site, also pharmascore empirical based energy function was used for rescoring to select the ligands that afford pharmacological interactions with target proteins, and use the ligand preferences to eliminate the ligands that violate the electrostatic or hydrophilic constraints. Finally, poses were visualized while using a discovery studio ligand interaction visualizer [32].

### 2.11. Statistical Analysis

All of the experiments were performed in triplicates. The data were expressed as mean ± standard deviation (SD) (*n* = 7). Statistical analyses were carried out while using SPSS software package (version 24.0., Armonk, NY, USA: IBM Corp.) and performed using one-way ANOVA, followed by L.S.D test and Duncan post-hoc test. Statistical significance* was set at *p* ≤ 0.05 and values at *p* ≤ 0.005 were considered to be statistically highly significant**.

## 3. Results and Discussion

### 3.1. Antioxidant Activities and Liver Function Parameters

Carbon tetrachloride is a well settled model for hepatoprotective studies [33]. The GSH level and total antioxidant capacity (TAC) were significantly increased in all of the pretreated groups up to a near normal value compared to CCl_4_ intoxicated rats, with superior activity being exhibited by the defatted ethanolic extract of *C. oliviforme* L. (Oe) at the higher dose level (500 mg/kg), as shown in Table 1. The defatted ethanolic extracts of both plants were found to be more efficient relative to the corresponding petroleum ether extracts, which was probably due to its phenolic components. The antioxidant properties of the tested extracts may be mediated by GSH regeneration from its oxidized form. A similar assertion was previously reported for *C. albidum* G. [34]. The etahnolic extract of *C. oliviforme* (Oe) could thus be considered as the most effective in reversing the harmful effect of CCl_4_-liver injury on ALT and AST raised levels when compared to the standard hepatoprotective drug, Silymarin with relative potencies 1.51 and 2.22, respectively (Table 2). The raised activity of serum transaminases in CCl_4_ intoxicated rats, as observed in the present study, can be attributed to the damaged structural integrity of liver cells and leakage of AST and ALT enzymes into circulation [35,36]. The tendency of these marker enzymes to return to near normalcy indicates hepatocyte regeneration [37]. In cases of high risk of delayed onset hepatitis-like reactions, serum ALT monitoring would be of little value unless, antioxidant activity, histopathological, and genetic expression level is confirming.

The reduction in serum total protein and albumin levels is a sign of severe liver injury induced by CCl_4_ that causes the disruption and disassociation of polyribosomes of endoplasmic reticulum, leading to a slump in protein biosynthesis. The protective effect of all the extracts on liver albumin and total protein levels, when compared to the positive control, might be related to endoplasmic reticulum stabilization (Table 2) [38].

### 3.2. Effect on Lipid Peroxidation and Lipid Profile

**Figure 1:**
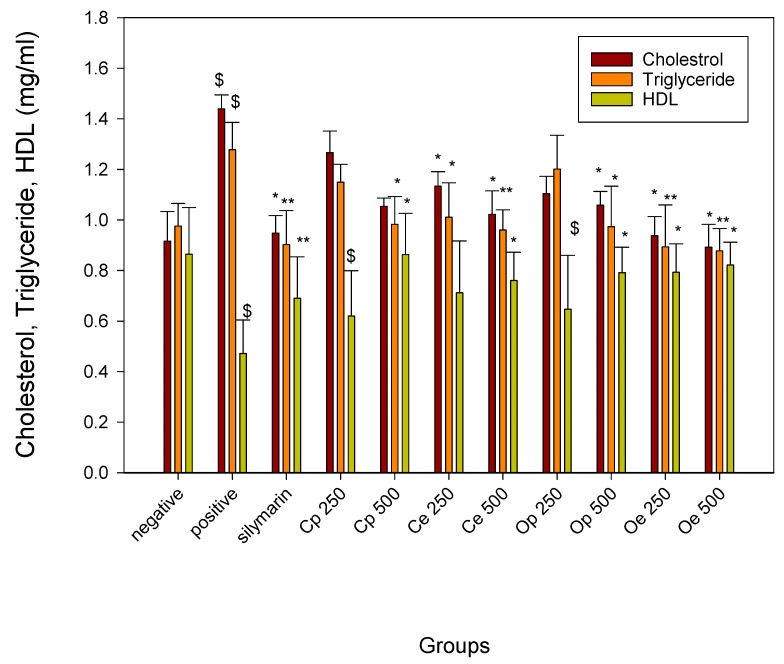
Effect on lipid profile observed after treatment with petroleum ether (Cp & Op) & defatted ethanolic (Ce & Oe) extracts of *C. cainito* L. and *C. oliviforme* L. leaves, respectively, in CCL_4_-induced liver injury model in rats. * Statistically significant as compared to control positive (*p* < 0.05), ** highly Statistically significant as compared to control positive (*p* < 0.005), ^$^ Statistically significant compared to control negative at *p* < 0.05.

Liver plays a fundamental role in the metabolism of phospholipids. The elevated MDA values in CCl_4_ intoxicated rats were significantly decreased in all of the pretreated groups in a dose dependent manner. The ethanolic extract of *C. oliviforme* (Oe), at the higher dose level (500 mg/kg), was found to be the most effective extract in terms of lipid peroxidation inhibition (Table 1). A marked decrease in triglyceride and total cholesterol levels and an increase in HDL level were recorded in liver-injured rats upon treatment with both the petroleum ether and defatted ethanolic extracts of the leaves of the two plants, as shown in (Figure 1), indicating a significant protective effect on liver through managing proper lipid metabolism. Noteworthy is that the defatted ethanolic extract (Oe) at a dose of 500 mg/kg was found to be the most pronounced extract in restoring the altered lipid profile due to CCl_4_-injury by decreasing cholesterol, triglyceride levels, and increasing HDL level with comparable potency to silymarin; 1.06, 1.03, and 1.19, respectively. The injection of CCl_4_ caused a significant disruption to phospholipids metabolism, an increase in TC, TG, and a significant decrease in HDL levels. The increase in cholesterol level might be due to an increased esterification of fatty acids, inhibition of fatty acid β-oxidation, and decreasing cell lipids excretion [39]. From another point of view, the pretreatment of liver-damaged rats with defatted ethanolic or petroleum ether extracts of two plants might inhibit the harmful effects of free radicles and prevent cell membrane oxidation. This action is expected to decrease acetate transportation to liver cells, which results in a decrease in cholesterol, triglycerides, and free fatty acid synthesis. As a synergy, increasing HDL levels by the plant extracts is an action that is expected to mediate acetate breakdown by the liver and, hence, decrease the lipid levels [40].

### 3.3. Histopathological examination

**Figure 2:**
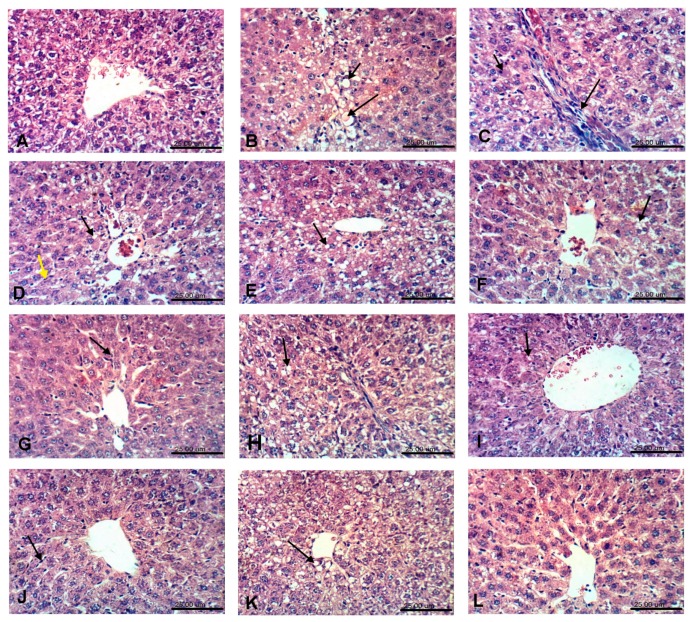
Photomicrographs of the tested extracts effect on liver tissues in CCl_4_-induced liver injury model in rats (H & E × 400); (**A**): Liver of normal rat from control –ve group showing apparent normal hepatic lobule. (**B**): Liver of untreated rat from control +ve group showing stenosis (small arrow). (**C**): Liver of untreated rat from control +ve group showing necrosis of sporadic hepatocytes (small arrow) and strands of fibroblasts proliferation (large arrow). (**D**): Liver of rat from standard group (silymarine) showing Kupffer cells activation (yellow arrow) and fatty change of some hepatocytes (large arrow). (**E**): Liver of rat from Cp 250 group showing fatty change of hepatocytes. (**F**): Liver of rat from Cp 500 group showing slight vacuolation of sporadic hepatocytes. (**G**): Liver of rat from Ce 250 mg/kg group showing Kupffer cells activation. (**H**): Liver of rat from Ce 500 mg/kg group showing very slight cytoplasmic vacuolization of hepatocytes. (**I**): Liver of rat from Op 250 mg/kg group showing slight vacuolization of sporadic hepatocytes. (**J**): Liver of rat from Op 500 group showing Kupffer cells activation. (**K**): Liver of rat from Oe 250 mg/kg group showing very slight vacuolization of hepatocytes. (**L**): Liver of rat from Oe 500 mg/kg group showing no histopathological changes.

**Figure 3:**
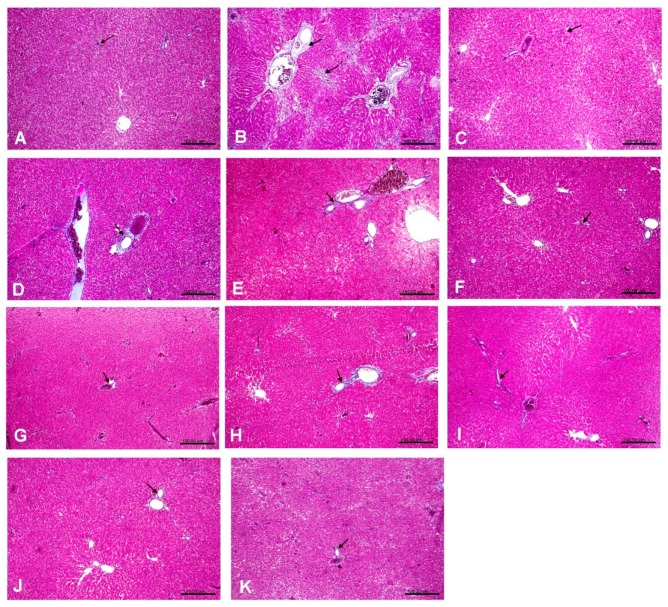
Photomicrographs of the tested extracts effect on liver tissues in CCl_4_-induced liver injury model in rats (Masson’s Trichrome stain × 10); arrows: reaction for collagen fibers, (**A**): Liver of normal rat from control –ve group showing normal (no) histochemical reaction for collagen fibers. (**B**): Liver of untreated rat from control +ve group showing strong positive histochemical reaction for collagen fibers. (**C**): Liver of rat from standard group (silymarin) showing weak positive histochemical reaction for collagen fibers. (**D**): Liver of rat from Cp 250 mg/kg group showing moderate positive histochemical reaction for collagen fibers. (**E**): Liver of rat from Cp 500 mg/kg group showing moderate positive histochemical reaction for collagen fibers. (**F**): Liver of rat from Ce 250 mg/kg group showing weak positive histochemical reaction for collagen fibers. (**G**): Liver of rat from Ce 500 mg/kg group showing weak positive histochemical reaction for collagen fibers. (**H**): Liver of rat from Op 250 mg/kg group showing moderate positive histochemical reaction for collagen fibers. (**I**): Liver of rat from Op 500 mg/kg group showing weak positive histochemical reaction for collagen fibers. (**J**): Liver of rat from group Oe 250 mg/kg showing weak positive histochemical reaction for collagen fibers. (**K**): Liver of rat from Oe 500 mg/kg group showing normal histochemical reaction for collagen fibers.

**Figure 4:**
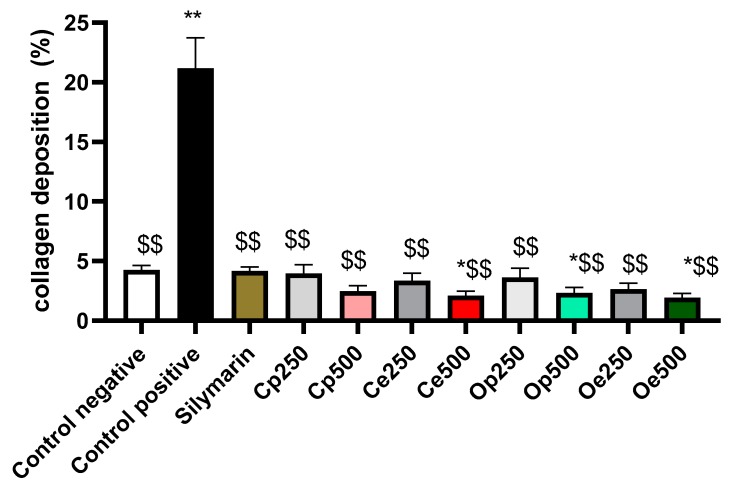
The measurement of the collagen deposition percentage of the tested extracts on liver tissues in CCl4-induced liver injury model in rats (Masson’s Trichrome stain × 10); determined using Image J software (http://rsbweb.nih.gov/ij/download.html). * Statistically significant as compared to control negative (P < 0.05), ** highly Statistically significant as compared to control negative (P < 0.005). ^$$^ Statistically significant compared to control positive at *p* < 0.005.

Photomicrographs of the liver tissues that were obtained from CCl_4_-intoxicated rats revealed that CCl_4_ induced cytoplasmic vacuolization and focal hepatic necrosis and mononuclear inflammatory cells infiltration. The extent of CCl_4_-induced necrosis was evaluated by assessing the morphological changes in the liver sections that were stained with hematoxylin and eosin (H & E) (Figure 2), and Masson’s trichrome stain (Figure 3). The effect of the tested extracts on collagen deposition percentage on Masson’s Trichrome stained (10×) liver tissues were determined while using Image J software (http://rsbweb.nih.gov/ij/download.html, National Institutes of Health and the Laboratory for Optical and Computational Instrumentation LOCI, University of Wisconsin, WI, USA) (Figure 4). The former changes were in agreement with the previously reported studies [41]. The pretreatment with the ethanolic extracts of both species at a dose of 500 mg/kg only showed slight vacuolization of hepatocytes and either week or negative histochemical reaction for collagen fibers indicating the protective potential of the extracts to hepatocytes. The defatted ethanol extract of *C. oliviform* (Oe) showed the best protective effect on liver. The metabolism of CCl_4_ in the liver results in the production of free radicles and initiates oxidative stress, which contributes to progression of liver injury. Oxidative stress will further promote the production of inflammatory cytokines, induce the necrosis of hepatocytes, and promote the progression of hepatic fibrogensis [42]. The hepatoprotective effect of the extracts might be attributed to its high phenolic and flavonoid contents.

### 3.4. In-Vivo Genetic Profiling

#### 3.4.1. DNA Laddering Assay

**Figure 5:**
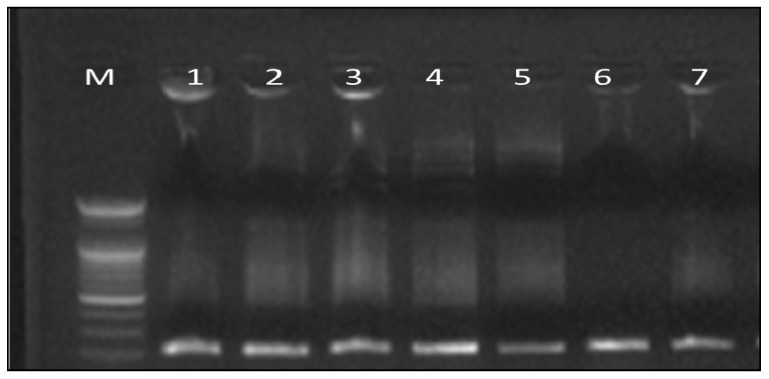
The electrophoresis of DNA fragments isolated from livers of treated rats. M refers to 100 bp DNA ladder, lane 1: control negative group; lane 2: control positive group; lane 3: silymarine group; lane 4: Ce 500 mg/kg group; lane 5: Cp 500 mg/kg group; lane 6: Oe 500 mg/kg group; lane 7: Op 500 mg/kg group.

**Figure 6:**
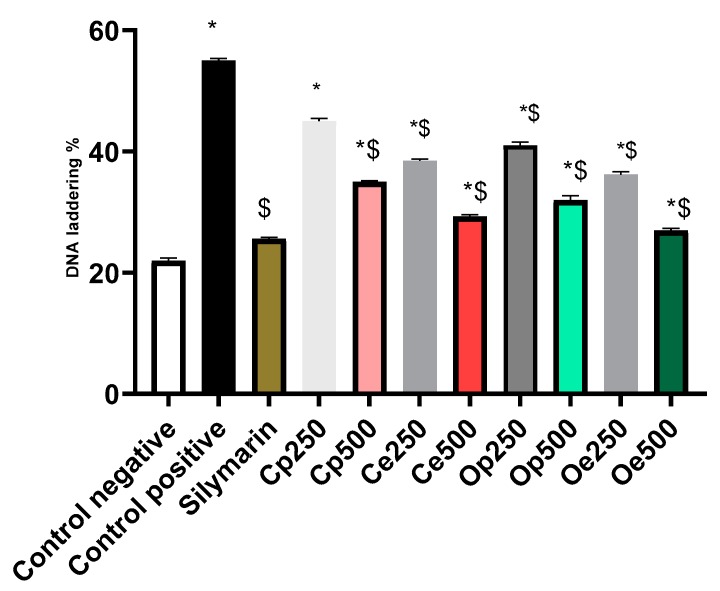
The DNA fragmentation percentage observed upon treatment with petroleum ether (Cp & Op) & defatted ethanolic (Ce & Oe) extracts of leaves. (Mean ± SE, *n* = 7). * indicates significant differences compared to control negative and $ indicates significant differences as compared to control positive (*p* < 0.05).

The CCl_4_-intoxicated rats were significantly affected and showed markedly elevated DNA fragmentation percentage. Administration of either petroleum ether or ethanolic plant extracts showed apparent protective effect denoted by a significant inhibition in DNA fragmentation%, reaching 27, 29.3, 32, and 35% for *C. oliviforme* (Oe), *C. canitio* (Ce) ethanolic extracts, and *C. oliviforme* (Op), *C. canitio* (Cp) petroleum ether extracts, respectively (Figure 5 and Figure 6). The ethanolic extract of the leaves of *C. oliviforme* (Oe) was the most effective relative to the standard hepatoprotective drug, silymarin (relative potency, 0.95).

#### 3.4.2. Measurement of mRNA Expression Level of TGF-β

**Figure 7:**
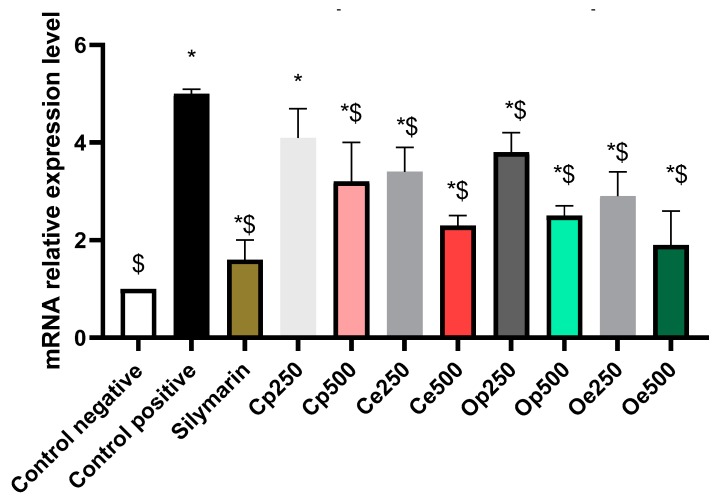
The relative fold quantitation of transforming growth factor-β (TGF-β) m-RNA; The mRNA expression level of TGF-β among treated groups. (mean ± SE, *n* = 7). *indicates significant differences compared to control negative and $ indicates significant differences compared to control positive (*p* < 0.05).

The mRNA level of the gene encoding TGF-β was measured while using qPCR. The results revealed that CCl_4_ intoxicated rats had marked increase in the TGF-β transcription level. Nevertheless, a significant reduction in TGF-β transcription level was observed in the treated groups, when compared to the positive control group. The values showed to be 1.9, 2.3, 2.5, and 3.2 for Oe, Ce, Op, and Cp, respectively (Figure 7), indicating the superior potency of *C. oliviforme* ethanolic extract (Oe) (potency relative to silymarin; 0.91).

In response to CCl_4_ treatment, an up-regulated expression of TGF-β, the indicator of early fibrosis via ROS (reactive oxygen species) generation, was recorded [43]. The cascade process triggers inflammation, which leads to hepatocytes damage and liver fibrogenesis [44]. This protective effect of the plant extracts might be attributed to the presence of phenolic compounds, which were proven to exert a potent antioxidant effect that diminished the adverse effect that is provoked by CCl_4_ [45]. Several studies showed that inhibition of TGF-β signaling pathways displays antifibrotic effects in experimental models [46]. Our results, in accordance with the previously estimated lipid profile, antioxidant, and histopathological studies, revealed that the leaf extracts of both plants could attenuate liver fibrosis. 

### 3.5. Extractives Yield and Phenolic Composition

Phenolic compounds have attracted a great interest in drug discovery due to their potential antioxidant activity. The total phenolic content (TPC) of *C. oliviforme* extract (Oe) was found to be 1.5 times as much as that of *C. cainito* (Ce) (375.66 ± 0.37 vs. 267.69 ± 0.92 μg GAE/mg extract). Although, the TFC of the latter was much higher (by about five times, reaching 56.47 ± 0.001 μg QE/mg) than that of the former (10.98 ± 0.061 μg QE/mg extract). The protective activity of these extracts against CCl_4_-induced hepatotoxicity is obviously associated with their phenolic components. The lack of information regarding those of *C. oliviforme* leaves besides the more pronounced activity of its ethanol extract (Oe) necessitated deeper investigation of its composition.

### 3.6. Phenolic Isolation

**Figure 8:**
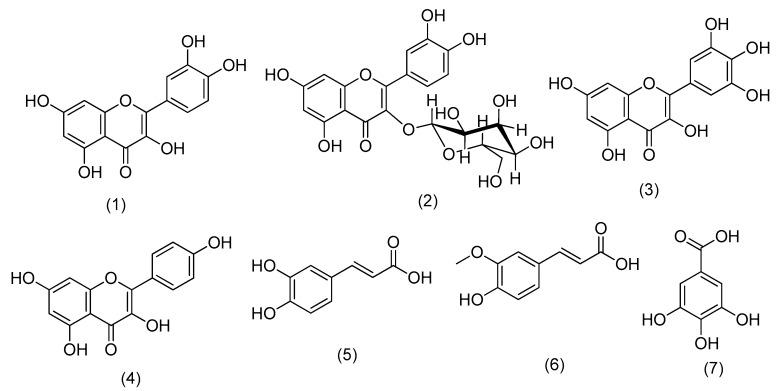
Isolated compounds from ethyl acetate extract of *C. oliviforme* L. leaves, Quercetin (**1**), Isoquercitrin (**2**), Myricetin (**3**), Kaempferol (**4**), Caffeic acid (**5**), trans-Ferulic (**6**), and Gallic acid (**7**).

Seven known phenolic compounds were isolated from the ethyl acetate fraction of *C. oliviforme*. The isolated compounds were identified via physicochemical examination and spectral analysis (UV, ^1^H NMR, and ^13^C NMR) and by comparison with reported data. They included four flavonols: quercetin (1), isoquercitrin (2), myricetin (3), and kaempferol (4); in addition to three phenolic acids namely, caffeic acid (5), trans-ferulic acid (6), and gallic acid (7) (Figure 8). As far as the available literature is concerned, this is the first report on isolation of these compounds from the leaves of *C. oliviforme* L., although being reported from other members of the genus *viz*., *C. albidum* G., *C. cainito* L., *C. flexuosum* Mart. and *C. marginatum* Hook. & Arn. [47,48,49]. The reported antioxidant activity is suspected to be due to isolated phenolic acids (caffeic, trans-ferulic, and gallic acids) and flavonol derivatives (3 aglycones viz, kaempferol, quercetin, myricetin, and isoquercitrin).

### 3.7. Molecular Study of Isolated Compounds (1- 7) on Gene Expression of TGF-β1

**Figure 9:**
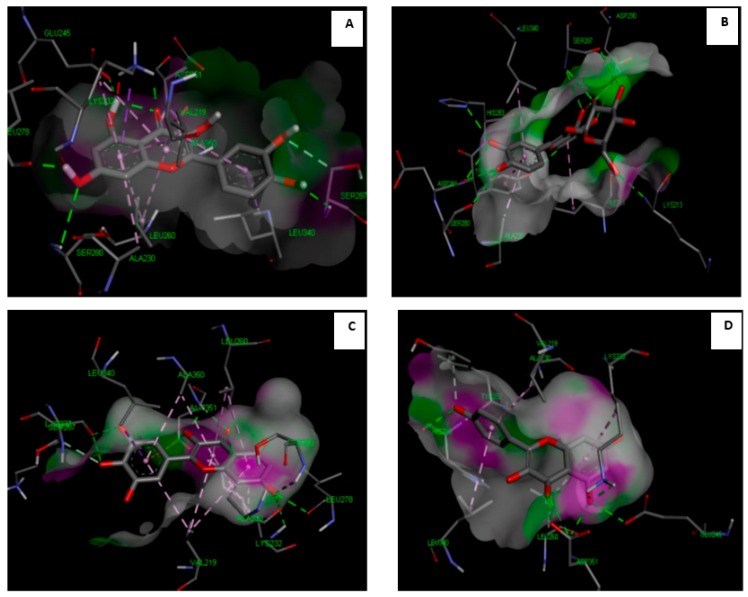
Molecular interaction of quercetin (**A**), isoquercitrin (**B**), myricetin (**C**), and kaempferol (**D**) with TGF-β1 receptors (PDB ID: 2QD9).

**Figure 10:**
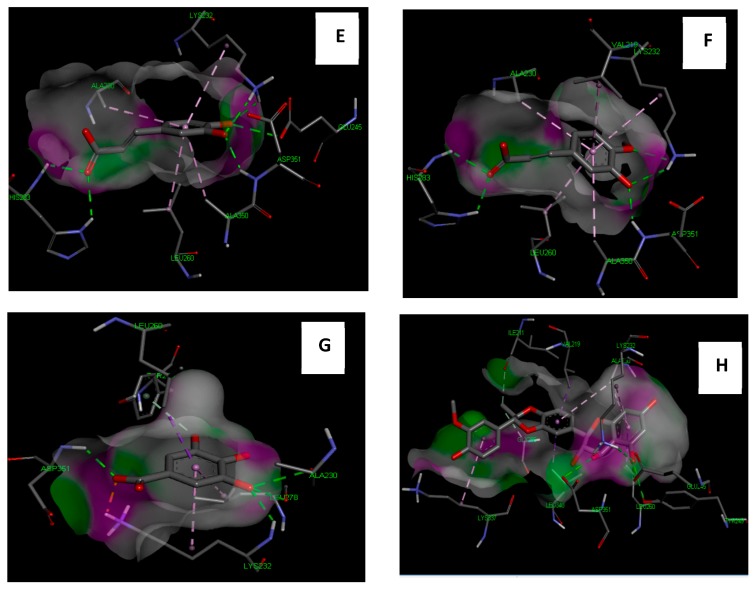
Molecular interaction of caffeic acid (**E**), trans-ferulic acid (**G**), gallic acid (**H**), and silibinin (**I**) with TGF-β1 receptors (PDB ID: 2QD9).

**Figure 11:**
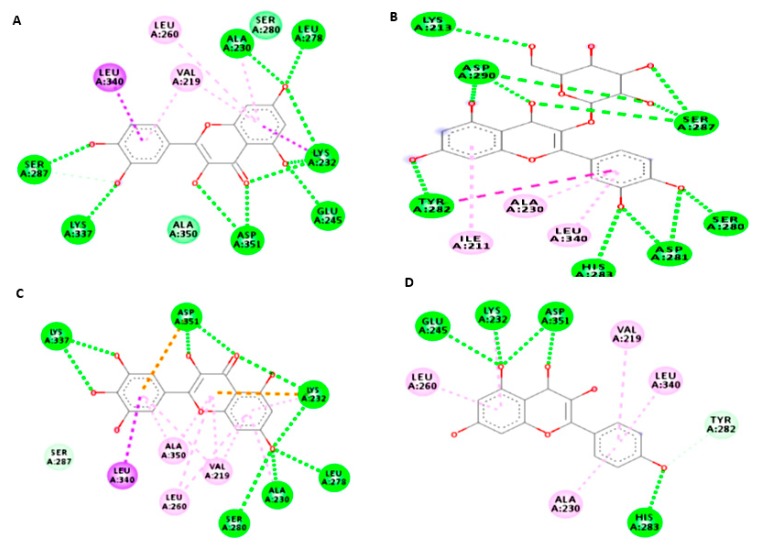
Ligand interaction diagrams of isolated compounds with TGF- β1. Quercitin (**A**), isoquercitin (**B**), myricetin (**C**), and kaempferol (**D**).

**Figure 12:**
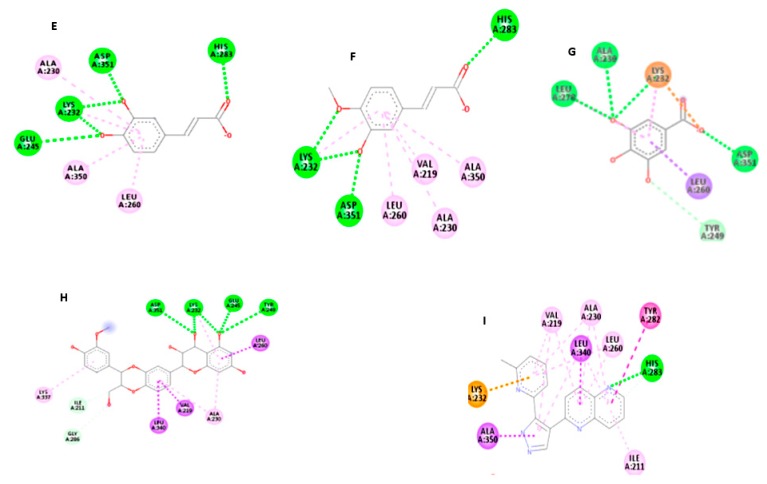
Ligand interaction diagrams of isolated compounds with TGF- β1; caffeic acid (**E**), trans-ferulic acid (**F**), gallic acid (**G**), Silibinin (**H**), and co-crystallized ligands (**I**).

Molecular docking study indicated that the compounds could bind to the active site properly in comparison to the co-crystallized ligand and silibinin, a known hepatoprotective compound (Figure 9, Figure 10, Figure 11 and Figure 12). In the context of the total energy that is required for binding, the co-crystallized ligand achieved -112 kcal/mole, while all of the investigated compounds could achieve comparable or lower binding energy except for gallic acid, trans-ferulic acid, and caffeic acid, as presented in (Table 3). Therefore, it could be assumed that flavonol-type flavonoids could be a promising lead compound for development of new inhibitors for such therapeutic targets for prevention and treatment of related hepatic conditions [50,51].

## 4. Conclusions

Our results clearly demonstrated that both *C. cainito* and *C. oliviforme* leaf extracts significantly alleviated CCl_4_-induced oxidative stress and liver damage, as evidenced by histopathological liver fibrosis changes and a marked hepatoprotective effect that are denoted by significant reduction in TGF-β transcription level, as well as significant inhibition in DNA laddering fragmentation. The ethanol extract of the defatted leaves of *C. oliviforme* could be favored over that of *C. cainito* due to its relatively higher yield and phenolic content, together with its more pronounced activities. Moreover, six compounds were isolated from *C. oliviforme* leaves, and the in silico studies suggest that flavonol-type flavonoids exhibited their hepatoprotective effect due to their ability to interact with TGF-β1. In conclusion, our in-vivo and in-silico study confirms the inhibitory effect of these compounds. However, extensive clinical trials must be performed to support all of the evaluated bioactivities and facilitate an implementation of these promising herbals in pharmaceutical formulations.

## Figures and Tables

**Table 1:** Antioxidant activity of *C. cainito* and *C. oliviforme* petroleum ether and defatted ethanolic extracts (250, 500 mg/kg. b.wt) in CCL_4_-induced liver injury model in rats.

Group	Reduced Glutathione (GSH)	Total Antioxidant Activity	Lipid Peroxidase (Malondialdehyde)
	mg/dL	mM/L	nmol/mL
Control negative	25.8 ± 2.05 *	2.25 ± 0.02 **	5.90 ± 0.46 **
Control positive	15.3 ± 1.04 ^$^	1.07 ± 0.10 ^$$^	12.23 ± 0.81 ^$$^
Silymarin	26.1 ± 1.32 **	1.88 ± 0.02 **	5.35 ± 0.35 **
Cp 250	20.8 ± 2.01	1.55 ± 0.20 ^$^	7.86 ± 0.64 **
Cp 500	24.4 ± 2.07 *	1.77 ± 0.14 *	6.15 ± 0.60 **
Ce 250	22.8 ± 2.14 *	1.89 ± 0.15 **	6.26 ± 0.54 **
Ce 500	25.2 ± 2.18 **	2.05 ± 0.04 **	5.97 ± 0.38 **
Op 250	22.2 ± 2.17 *	1.92 ± 0.08 **	6.74 ± 0.53 **
Op 500	25.0 ± 1.52 **	1.97 ± 0.07 **	6.37 ± 0.36 **
Oe 250	23.9 ± 2.25 *	1.95 ± 0.05 **	5.49 ± 0.30 **
Oe 500	26.3 ± 1.45 **	2.06 ± 0.04 **	4.88 ± 0.42 **

Cp; *C. canito* (pet. ether extract), Ce; *C. canito* (ethanol extract), Op; *C. oliviform* (pet.ether extract), Oe; *C. oliviform* (ethanol extract), *n* = 7, mean ± SD, SD; standard deviation, * Statistically significant compared to control positive at *p* < 0.05, ** Statistically significant compared to control positive at *p* < 0.005. ^$^ Statistically significant compared to control negative at *p* < 0.05. ^$$^ Statistically significant compared to control negative at *p* < 0.005.

**Table 2:** Effect of *C. cainito* L. and C. *oliviforme* L. petroleum ether and defatted ethanolic extracts (250, 500 mg/kg. b.wt) on liver function in CCL_4_-induced liver injury model in rats.

Group	AST	ALT	Total Protein	Albumin
	IU/L		g/dL	
Control negative	41.0 ± 1.24 *	31.3 ± 3.26	7.57 ± 0.65 *	3.915 ± 0.17 *
Control positive	70.4 ± 4.93 ^$^	48.6 ± 4.25 ^$^	3.25 ± 0.94 ^$^	1.23 ± 0.08 ^$^
Silymarin	41.9 ± 2.13 **	27.6 ± 3.51 *	7.49 ± 1.27 *	3.03 ± 0.42 *
Cp 250	62.2 ± 4.75 *	32.5 ± 4.74 *	7.1 ± 1.57 *	3.84 ± 0.37 *
Cp 500	46.5 ± 1.12	29.8 ± 5.53 *	6.98 ± 1.06 *	3.48 ± 0.19 *
Ce 250	23.8 ± 1.02 ^**,$^	21.7 ± 1.96 *	7.23 ± 0.98 *	3.29 ± 0.15 *
Ce 500	20.3 ± 1.52 ^**,$^	24.2 ± 1.60 *	7.55 ± 0.43 *	3.16 ± 0.19 *
Op 250	37.5 ± 2.10 *	24.8 ± 4.57 *	7.41 ± 0.31 *	3.17 ± 0.19 *
Op 500	29.0 ± 1.42 ^**,$^	21.9 ± 1.56 *	7.74 ± 0.71 *	3.20 ± 0.26 *
Oe 250	22.38 ± 1.48 ^**,$^	21.9 ± 2.09 *	7.58 ± 0.74 *	3.16 ± 0.20 *
Oe 500	19.4 ± 1.60 ^**,$^	20.5 ± 1.13 *	7.70 ± 0.62 *	3.10 ± 0.20 *

Cp; *C. canito* (pet. ether extract), Ce; *C. canito* (ethanol extract), Op; *C. oliviform* (pet. ether extract), Oe; *C. oliviform* (ethanol extract). *n* = 7, mean ± SD, SD; standard deviation, * Statistically significant compared to control positive at *p* < 0.05, ** Statistically significant compared to control positive at *p* < 0.005. ^$^ Statistically significant compared to control negative at *p* < 0.05.

**Table 3:** The Pharmascore, total energy and interaction with amino acid residues from docking the isolated compounds (1–7) and standard silibinin in the active site of TGF-β1 in comparison with the co-crystalized ligand.

Compounds	Human TGF-β1				
	Pharmascore	Interaction with Key Amino Acid	Total Binding Energy	VDW	H Bond
Quercetin	−149.1	LEU-260, LEU-340, LYS-232, VAL-219, ASP-351	−116.7	−83.7005	−33.0111
Isoquercitrin	−158.4	LEU-260, LEU-340, VAL-219	−150.5	−108.313	−42.209
Myricetin	−152	LEU-260, LEU-340, LYS-232, VAL-219, ASP-351	−118.5	−87.6131	−30.8507
Kaempferol	−141.7	LEU-260, LEU-340, LYS-232, VAL-219, ASP-351	−108.2	−81.402	−26.8215
Caffeic acid	−106.9	LEU-260, LEU-340, LYS-232, VAL-219, ASP-351	−78.7	−54.8976	−22.2599
Trans-Ferulic acid	−112.7	LEU-260, LEU-340, LYS-232, VAL-219, ASP-351	−84.4	−65.2541	−17.6243
Gallic acid	−113	LEU-260, LYS-232, VAL-219, ASP-351.	−86.7	−63.9845	−25.2021
Silbinin	−173.4	LEU-260, LEU-340, LYS-232, VAL-219, ASP-351	−145.1	−121.657	−23.4008
Reference inhibitor (co-crystallized ligand)	−127.7	LEU-260, LEU-340, LYS-232, VAL-219	−112	−102.822	−9.14506

## Abbreviations

GAE: gallic acid equivalent; GSH, reduced glutathione; H & E, hematoxylin and eosin; HDL, high-density lipoproteins; MDA, malondialdehyde; QE, quercetin equivalent; TAC, total antioxidant capacity; TC, total cholesterol; TG, triglycerides; TGF-β, transforming growth factor-β, Op petroleum ether extract of *C. oliviforme* L., Cp petroleum ether extract of *C. cainito* L., Oe defatted ethanolic extract of *C. oliviforme* L., Ce defatted ethanolic extract of *C. cainito* L.
